# Does urinary cytology have a role in haematuria investigations?

**DOI:** 10.1111/bju.14459

**Published:** 2018-08-29

**Authors:** Wei Shen Tan, Rachael Sarpong, Pramit Khetrapal, Simon Rodney, Hugh Mostafid, Joanne Cresswell, Dawn Watson, Abhay Rane, James Hicks, Giles Hellawell, Melissa Davies, Shalom J. Srirangam, Louise Dawson, David Payne, Norman Williams, Chris Brew‐Graves, Andrew Feber, John D. Kelly, DETECT I trial collaborators, AN Sridhar, BW Lamb, F Ocampo, H McBain, K Baillie, K Middleton, H Knight, S Maher, B Pathmanathan, A Harmathova, S Pelluri, J Pati, A Cossons, C Scott, S Madaan, S Bradfield, N Wakeford, Dann, J Cook, M Cornwell, R Mills, Thomas, S Reyner, G Vallejera, P Adeniran, S Masood, N Whotton, K Dent, S Pearson, J Hatton, M Newton, E Hheeney, K Green, S Evans, M Rogers, K Gupwell, S ley, A Brown, J McGrath, N Lunt, P Hill, A inclair, A Paredes‐Guerra, B Holbrook, E Ong, H Wardle, D Wilson, A Bayles, R Fennelly, M Tribbeck, K Ames, J A Taylor, E Edmunds, J Moore, S Mckinley, T Nolan, A peed, A Tunnicliff, G Fossey, A Williams, M George, I Hutchins, R Einosas, A Richards, A Henderson, B Appleby, L Kehoe, L Gladwell, S Drakeley, J A Davies, R Krishnan, H Roberts, C Main, S Jain, J Dumville, N Wilkinson, J Taylor, F Thomas, K Goulden, C Vinod, E Green, C Waymont, J Rogers, A Grant, V Carter, H Heap, C Lomas, P Cooke, L Scarratt, T Hodgkiss, D Johnstone, J Johnson, J Allsop, J Rothwell, K Connolly, J Cherian, S Ridgway, M Coulding, H Savill, J Mccormick, M Clark, G Collins, K Jewers, S Keith, G Bowen, J Hargreaves, K Riley, A Rees, S Williams, S Dukes, A Goffe, R Mistry, J Chadwick, S Cocks, R Hull, A oftus, Y Baird, S Moore, S Greenslade, J Margalef, I Chadbourn, M Harris, P Clitheroe, S Connolly, S Hodgkinson, H Haydock, E Storr, L Cogley, S Natale, W Lovegrove, K Slack, D Nash, K Smith, J Walsh, A M Guerdette, M Hill, B Taylor, E Sinclair, M Perry, M Debbarma, D Hewitt, R Sriram, A Power, J Cannon, L Devereaux, A Thompson, K Atkinson, L Royle, J Madine, K MacLean

**Affiliations:** ^1^ Division of Surgery and Interventional Science University College London London UK; ^2^ Department of Urology University College London Hospital London UK; ^3^ Surgical and Interventional Trials Unit University College London London UK; ^4^ UCL Cancer Institute London UK; ^5^ Department of Urology Royal Surrey County Hospital Guildford UK; ^6^ Department of Urology James Cook University Hospital Middlesbrough UK; ^7^ Department of Urology East Surrey Hospital Redhill UK; ^8^ Department of Urology Worthing Hospital Worthing UK; ^9^ Department of Urology Northwick Park Hospital London UK; ^10^ Department of Urology Salisbury District Hospital Salisbury UK; ^11^ Department of Urology East Lancashire Hospital Blackburn UK; ^12^ Department of Urology Royal Bolton Hospital Bolton UK; ^13^ Department of Urology Kettering General Hospital Kettering UK

**Keywords:** biomarker, cytology, diagnosis, haematuria, urine, investigations, #utuc, #blcsm, #BladderCancer

## Abstract

**Objectives:**

To determine the diagnostic accuracy of urinary cytology to diagnose bladder cancer and upper tract urothelial cancer (UTUC) as well as the outcome of patients with a positive urine cytology and normal haematuria investigations in patients in a multicentre prospective observational study of patients investigated for haematuria.

**Patient and methods:**

The DETECT I study (clinicaltrials.gov NCT02676180) recruited patients presenting with haematuria following referral to secondary case at 40 hospitals. All patients had a cystoscopy and upper tract imaging (renal bladder ultrasound [RBUS] and/ or CT urogram [CTU]). Patients, where urine cytology were performed, were sub‐analysed. The reference standard for the diagnosis of bladder cancer and UTUC was histological confirmation of cancer. A positive urine cytology was defined as a urine cytology suspicious for neoplastic cells or atypical cells.

**Results:**

Of the 3 556 patients recruited, urine cytology was performed in 567 (15.9%) patients from nine hospitals. Median time between positive urine cytology and endoscopic tumour resection was 27 (IQR: 21.3–33.8) days. Bladder cancer was diagnosed in 39 (6.9%) patients and UTUC in 8 (1.4%) patients. The accuracy of urinary cytology for the diagnosis of bladder cancer and UTUC was: sensitivity 43.5%, specificity 95.7%, positive predictive value (PPV) 47.6% and negative predictive value (NPV) 94.9%. A total of 21 bladder cancers and 5 UTUC were missed. Bladder cancers missed according to grade and stage were as follows: 4 (19%) were ≥ pT2, 2 (9.5%) were G3 pT1, 10 (47.6%) were G3/2 pTa and 5 (23.8%) were G1 pTa. High‐risk cancer was confirmed in 8 (38%) patients. There was a marginal improvement in sensitivity (57.7%) for high‐risk cancers. When urine cytology was combined with imaging, the diagnostic performance improved with CTU (sensitivity 90.2%, specificity 94.9%) superior to RBUS (sensitivity 66.7%, specificity 96.7%). False positive cytology results were confirmed in 22 patients, of which 12 (54.5%) had further invasive tests and 5 (22.7%) had a repeat cytology. No cancer was identified in these patients during follow‐up.

**Conclusions:**

Urine cytology will miss a significant number of muscle‐invasive bladder cancer and high‐risk disease. Our results suggest that urine cytology should not be routinely performed as part of haematuria investigations. The role of urine cytology in select cases should be considered in the context of the impact of a false positive result leading to further potentially invasive tests conducted under general anaesthesia.

**Abbreviations**
CIS: carcinoma *in situ*
CTU: CT urogram
FISH: fluorescence *in situ* hybridization
IQR: interquartile range
NICE: National Institute for Health and Care Excellence
NMP22: nuclear matrix protein 22
NPV: negative predictive value
NVH: non‐visible haematuria
PPV: positive predictive value
RBUS: renal/bladder ultrasonography
UTUC: upper tract urothelial cancer
VH: visible haematuria

## Introduction

Cystoscopy with upper tract imaging is the recommended investigation when evaluating patients with haematuria to identify bladder cancer or upper tract cancer. Urinary cytology is a frequently used test and has been available in most hospitals since it was described by Papanicolaou and Marshall 1. Urine cytology has a high specificity and variable sensitivity (38–84%), even in high grade disease, and an even lower sensitivity for low grade bladder cancer (20–53%) 2. Hence, even with a high negative predictive value (NPV) of 92%, urinary cytology cannot be recommended as a standalone test 3.

There is no consensus among guideline bodies regarding the inclusion of urinary cytology for assessment of haematuria. The National Institute for Health and Care Excellence (NICE) bladder cancer guidelines do not specify investigations in patients with haematuria, but they do recommend that patients with a new diagnosis of bladder cancer should undergo one of the following: urine cytology; an alternative urinary biomarker test (such as UroVysion using fluorescence *in situ* hybridization [FISH], ImmunoCyt or nuclear matrix protein 22 [NMP22]); photodynamic diagnosis; or narrow‐band imaging 4. The AUA suggests that cytology may be useful for patients with persistent non‐visible haematuria (NVH) after a negative evaluation or those with carcinoma *in situ* (CIS) risk factors (irritative voiding, current/past tobacco use, chemical exposure) 5. Such inconsistent recommendations result in a variation in clinical practice.

The DETECT I study (ClinicalTrials.gov: NCT02676180) was a prospective observational study which recruited patients from 40 UK hospitals who underwent investigations for haematuria 6. Urinary cytology was performed as part of routine investigations for patients referred for investigation after a presentation of haematuria at one of nine centres. In the present paper, we report the diagnostic ability of urinary cytology to diagnose bladder cancer and upper tract urothelial cancer (UTUC) in patients with haematuria. A secondary aim of the study was to report the outcome of patients with positive urinary cytology after a normal cystoscopy and upper tract imaging.

## Methods

### Study Design

Between March 2016 and June 2017, patients were prospectively recruited from 40 hospitals in England. All patients were referred by their GP to secondary care after presentation of haematuria. Visible haematuria (VH) was defined as haematuria reported by the patient, while NVH was defined as a value of ≥1+ of blood on urine dipstick on ≥2 occasions 7. Men and women aged ≥18 years, who consented to undergo cystoscopy and upper tract imaging within 12 weeks from study registration, were eligible for inclusion. Patients who did not consent were excluded from the study. Verbal and written consent were obtained from all patients prior to cystoscopy.

The DETECT I full trial protocol has been previously reported 6. Study protocol was approved by the Health Research Authority‐North West Liverpool Central Research Ethics Committee on March 2016 (IRAS ID: 179245, REC reference: 16/NW/0150).

### Procedures

A medical history and physical examination were performed. All patients underwent flexible cystoscopy. The choice of upper tract imaging and use of urinary cytology was determined by local guidelines or physician preference. Upper tract imaging comprised renal/bladder ultrasonography (RBUS) and CT urogram (CTU). Where there was a suspicion of bladder cancer, patients underwent a subsequent transurethral resection of bladder tumour or bladder biopsy under anaesthesia. All urine samples collected for cytology were voided samples collected prior to cystoscopy. DETECT I was a pragmatic study and investigated the role of urinary cytology in routine practice; hence, central review was not performed. Urine samples were sent to the receiving laboratory, where they were centrifuged and a monolayer of cells was prepared on a glass slide. Cells were then stained with Papanicolaou staining and examined by microscopy by a cytopathologist.

### Outcome

Patient demographics, ethnicity and smoking history were recorded. The presence of bladder cancer was confirmed by histology according to TNM WHO classification 8. Cancer risk classification was defined based on European Association of Urology guidelines 9. The reference standard for the diagnosis of UTUC was by histopathological analysis.

Urinary cytology results were classified as (i) suspicious/consistent with neoplastic cells, (ii) atypical cells or (iii) negative for cancer. A positive/atypical urinary cytology was defined as a score of ≥3 on the Paris system for reporting of urinary cytology 10. Urine samples with inadequate cellular content were excluded from analysis. Analysis reporting the combined diagnostic performance of urinary cytology and imaging is determined based on the ability of either urinary cytology or imaging to detect bladder cancer or UTUC.

### Statistical Analysis

Continuous data were reported as descriptive statistics using mean, median, interquartile range (IQR) and 95% CI. Categorical variables were compared using the chi‐squared test and continuous variables were analysed using the *t*‐test. Normal distribution was assumed. Sensitivity, specificity, positive predictive value (PPV) and NPV were calculated for correct identification of bladder cancer or upper tract TCC. SPSS v22 (IBM Corp, Armonk, NY, USA) was used to perform all statistical analysis. *P* values <0.05 were taken to indicate statistical significance. This study was registered with ClinicalTrials.gov: NCT02676180.

## Results

Of the 3 556 patients recruited, urinary cytology was performed in 567 (15.9%) as a routine test in nine of the 40 participating hospitals. In all cases, urinary cytology was submitted in addition to cystoscopy and upper tract imaging. Patient demographics of the 567 patients are shown in Table 1. The median patient age was 67.7 years and 395 (69.7%) and 172 (30.3%) patients were investigated after a presentation of VH or NVH, respectively. In total, 39 bladder cancers (6.9%) and eight UTUCs (1.4%) were identified in the cohort. Previously, we reported an overall incidence of bladder cancer and UTUC of 8.0% and 0.7%, respectively in the entire cohort of 3 556 patients 11. The median (IQR) time interval between a positive urine cystoscopy to endoscopic tumour resection was 27 (21.3–33.8) days.

**Table 1:** Patient, cytology and histopathological characteristics (*N* = 567)

Variables	
Median (IQR) age, years	67.7 (55.6, 75.7)
Gender, *n* (%)	
Men	342 (60.3)
Women	225 (39.7)
Smoking history, *n* (%)	
Non‐smoker	240 (42.3)
Current smoker	87 (15.3)
Previous smoker	231 (40.7)
Not known	9 (1.6)
Type of haematuria, *n* (%)	
Visible	395 (69.7)
Non‐visible	172 (30.3)
Urine cytology, *n* (%)	
Inadequate cellular content/non‐diagnostic	13 (2.3)
Negative	512 (90.3)
Atypical	21 (3.7)
Suspicious/consistent with neoplastic cells	21 (3.7)
Bladder cancer, *n* (%)	39 (6.9)
Upper tract TCC, *n* (%)	8 (1.4)
Bladder cancer grade, *n* (%)	
G1	6 (15.4)
G2	14 (35.9)
G3	19 (48.7)
Concurrent CIS, *n* (%)	6 (15.4)
Bladder cancer stage, *n* (%)	
CIS	0 (0)
pTa	24 (61.5)
pT1	8 (20.5)
≥pT2	7 (17.9)

CIS, carcinoma *in situ*; IQR, interquartile range.

### Diagnostic Performance of Urine Cytology

**Figure 1:**
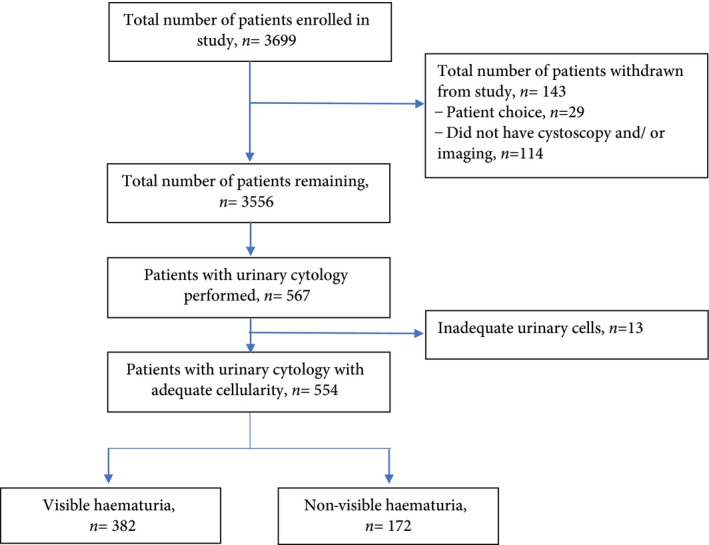
Flow diagram of patients recruited into study.

Thirteen urinary samples (2.3%) were excluded as a result of inadequate urinary cellular content for cytology analysis (Fig. 1). The overall accuracy of a positive/atypical urinary cytology for the diagnosis of bladder cancer or UTUC was: sensitivity 43.5%, specificity 95.7%, PPV 47.6% and NPV 94.9% (Table 2) with a receiver‐operating characteristic (ROC) of 0.713. The diagnostic ability of a positive/atypical urinary cytology to identify high‐risk disease was marginally better: sensitivity 57.7%, specificity 94.9%, PPV 35.7% and NPV 97.9%, with an ROC of 0.688 (Table 2). Selecting patients with VH only had a similar diagnostic performance (Table 2). Sub‐analysis of atypical urinary cytology suggests a low sensitivity of 6.0%, while a positive urinary cytology achieved a specificity of 98.4% with an ROC of 0.856 (Table 2). In total, 26 patients (52.3%) had a false‐negative result for urine cytology, of whom 21 had bladder cancers and five had UTUC. Bladder cancers missed according to grade and stage were as follows: four (19%) ≥ pT2, two (9.5%) G3 pT1, 10 (47.6%) G3/2 pTa and five (23.8%) G1 pTa. High‐risk cancer accounted for 38% of patients. No bladder cancer or UTUC was diagnosed based on a suspicious urinary cytology test alone. Stratifying patients according to smoking history did not change the performance of urinary cytology.

**Table 2:** Diagnostic accuracy of urinary cytology in isolation and urinary cytology in combination with CT urogram or renal/bladder ultrasonography to diagnose bladder cancer and upper tract urothelial cancer

Test	Patient cohort	Diagnostic accuracy, %				
		Sensitivity (95% CI)	Specificity (95% CI)	PPV (95% CI)	NPV (95% CI)	ROC
Positive/atypical urine cytology	All patients	43.5 (29.8–57.9)	95.7 (93.7–97.2)	47.6 (33.0–62.5)	94.9 (92.8–96.6)	0.713 (0.615–0.811)
	VH	44.2 (30.0–59.0)	94.7 (92.0– 96.7)	51.4 (35.6–67.0)	93.0 (90.0– 95.4)	0.722 (0.619–0.825)
	High risk bladder cancer	57.7 (38.7–75.3)	94.9 (92.8–96.6)	35.7 (22.4–50.7)	97.9 (96.3–98.9)	0.688 (0.567–0.769)
Positive urine cytology	All patients	38.2 (23.2–55.0)	98.4 (97.0–99.3)	61.9 (40.7–80.4)	95.9 (93.9–97.4)	0.856 (0.747–0.964)
Atypical urine cytology	All patients	6.0 (5.3–33.4)	96.7 (94.8–98.0)	19.0 (6.3–38.9)	95.9 (93.9–97.4)	0.570 (0.433–0.707
Positive/atypical urinary cytology or suspicious CTU suggestive of bladder cancer or UTUC	All patients	90.2 (78.8–96.9)	94.9 (91.9–97.0)	71.2 (58.0–82.2)	98.6 (96.7–99.6)	0.849 (0.773–0.924)
	VH	92.3 (81.2–98.0)	94.4 (91.1–96.8)	72.0 (58.7–83.1)	98.7 (96.8–99.7)	0.854 (0.778–0.930)
Positive/atypical urinary cytology or suspicious RBUS suggestive of bladder cancer or UTUC	All patients	66.7 (34.5–90.5)	96.7 (94.0–98.5)	42.9 (19.8–68.3)	98.8 (96.8–99.7)	0.708 (0.535–0.882)
	VH	66.7 (34.5–90.5)	96.6 (92.3–98.9)	60.0 (30.0–85.4)	97.4 (93.5–99.4)	0.787 (0.597–0.977)

CTU, CT urogram; NPV, negative predictive value; PPV, positive predictive value; RBUS, renal/bladder ultrasonography; ROC, receiver‐operating characteristic; UTUC, upper tract urothelial cancer; VH, visible haematuria.

### Outcome of Suspicious Urinary Cytology with Normal Cystoscopy and Upper Tract Imaging

Twenty‐two patients had a positive urinary cytology result despite a normal cystoscopy and upper tract imaging. Twelve patients (54.5%) had a further diagnostic procedure in the form of ureteroscopy with/without biopsy (*n* = 5) or interval cystoscopy (*n* = 7). No bladder cancer, ureteric or renal pelvis UTUC was identified. Five patients (22.7%) underwent repeat urinary cytology which was normal. Urinary cytology in two patients (9.1%) was reported as scanty mild atypia cells and ignored. A further three patients(13.6%) were lost to follow‐up. No patient had a subsequent diagnosis of cancer after further investigations. At the point of analysis, all patients had a minimum of 1‐year follow‐up.

### Diagnostic Performance of Urinary cytology with Upper Tract Imaging

The combination of urinary cytology with urinary tract imaging significantly increased the diagnostic performance to detect bladder cancer and UTUC compared with cytology alone. (Table 2). The combination of urinary cytology with CTU (sensitivity: 92.3, specificity: 94.9%) was superior to urinary cytology with RBUS (sensitivity: 66.7%, specificity: 96.7%). By comparison, CTU alone achieved a diagnostic performance of sensitivity 80.5%, specificity 97.0%, PPV 79.3% and NPV 97.2%, while RBUS had a sensitivity of 50.7%, a specificity of 99.3%, a PPV 84.3% and an NPV of 96.5% 12.

## Discussion

In the present paper, we report the diagnostic performance of urinary cytology to detect bladder cancer and UTUC in a multi‐centre prospective haematuria study. To our knowledge, this is the first multi‐centre UK study evaluating the ‘real‐world’ diagnostic accuracy of urinary cytology in the haematuria setting. Eight of the nine hospitals routinely performing urinary cytology in this study were district general hospitals. The prospective, structured design represents a strength of this study and the multi‐centre recruitment allows results to be generalized to the wider UK population. The diagnostic ability of urinary cytology was poor, even for diagnosis of high grade bladder cancer and regardless of risk group stratification, such as by those with VH.

There have been two historic single‐centre reports on the role of urinary cytology in the haematuria setting. Hofland et al. 13 reported that urinary cytology successfully identified cancer that was missed on cystoscopy or imaging in 0.2% (*n* = 2), while the study by Mishriki et al. 14 suggested that 0.07% (*n* = 2) of patients benefited from urinary cytology. In the present study, urinary cytology did not detect additional cancers identified by urinary cytology or imaging and the results suggest that routine urinary cytology has no added benefit for the assessment of haematuria.

Table 3 summarizes the recommendation of the AUA, NICE, BAUS (subsequently replaced by NICE), National Comprehensive Cancer Network, Canadian, Dutch and Japanese Urology Associations [4, 5, 15, 16, 17, 18, 19]. With the exception of the previous BAUS haematuria recommendations, all other guidelines recommend the use of urinary cytology in selected patients presenting with haematuria; however, there is no consistency, and the recommended patient groups that may benefit from urinary cytology varies among the guidelines 20.

**Table 3:** Comparison of recommendations on the use of urinary cytology

AUA 5	Cytology not recommended for asymptomatic NVH. In patients with persistent NVH after a negative evaluation or those with CIS risk factors (irritative voiding, current/past tobacco use, chemical exposure) cytology may be useful. No comment for VH
CUA 15	All haematuria patients should undergo cytology. Those with negative investigations should undergo urinary cytology in conjunction with urine analysis and blood pressure checks at 6, 12, 24 and 36 months. No comment for VH
BAUS 16	Cytology not part of VH or NVH investigations
NICE 4	Role of cytology not commented on for initial investigations. Cytology/urinary biomarkers or photodynamic diagnosis/narrow band imaging in patients with suspected bladder cancer
NCCN 17	Role of cytology not commented on for initial investigations. Consider cytology for suspected bladder cancer
JUA 18	Cytology recommended for VH. NVH without risk factors should be subject to renal tract ultrasonography or cytology
DAU 19	Cytology recommended in VH patients of any age or NVH at age >50 years after a negative evaluation

AUA, American Urology Association; BAUS, British Association of Urological Surgeons; CIS, carcinoma *in situ*; CUA, Canadian Urology Association; DAU, Dutch Association of Urology; JUA, Japan Urology Association; NICE, National Institute for Health and Care Excellence; NCCN, National Comprehensive Cancer Network; NVH, non‐visible haematuria; VH, visible haematuria.

While urinary cytology has a high specificity, the sensitivity of urinary cytology can range from 12% to 85% 2, 21. The proportion of high grade tumours, inter‐observer variability, sample preparation and differences in urine collection methods can explain this wide variation. Ideally, urine samples collected for cytology should include three daily mid‐morning or random samples and be transferred to the receiving laboratory in a timely manner 22. Where long delays are expected, an equal volume of 50% alcohol should be added to allow prompt fixation. Multiple urine voided samples have been shown to increase the sensitivity from 44% to 67% in a retrospective single‐institution study 23; however, in clinical practice, this is rarely performed. Patients are often seen in a busy one‐stop haematuria clinic and, because of time constraints, only one voided urine sample is collected and used for both urine analysis and urine cytology.

Over time, different reporting criteria have been used when reporting urinary cytology 24, but none of these criteria has gained widespread acceptance, resulting in significant variation in reporting. In addition, there is significant intra‐observer variability between cytopathologists, even when the same reporting criteria are used 24, 25. Central review of 652 urinary cytology specimens showed a κ coefficient of between 0.36 and 0.45 for non‐tertiary institutions 25.

In addition, a report of ‘atypical’ urinary cytology represents a diagnostic conundrum. There is no consensus on the exact classification of atypical urine cytology. Published reports suggest that up to 23.2% of urinary cytology results are categorized as atypical 26. The prognostic value of atypical urinary cytology is debatable. A retrospective analysis of 1320 patients with atypical urinary cytology suggests that 21% of cases will develop malignancy with a mean follow‐up of 155 days, although others have questioned the significance of the atypical category 26, 27.

The cost of urinary cytology is estimated to be £114.55 (2012 adjusted cost) based on a Health Technology Assessment estimate 28. Flexible cystoscopy and imaging are estimated to cost £401.88 and £83.85, respectively, suggesting that urinary cytology, if performed, cost nearly 20% of the cost of haematuria investigations. No guideline body recommends that urinary cytology or any other urinary biomarkers should replace cystoscopy, and direct visualization of the bladder is recommended in patients with haematuria. Other commercially available urinary biomarkers, such as fluorescence *in situ* hybridization, NMP22, ImmunoCyst and Cxbladder, achieve a sensitivity of 57–82% and a specificity of 74–88%, which will miss a substantial number of bladder cancers with a high risk of false‐positive results 29. The requirement for cystoscopy and upper tract imaging makes the need for cytology redundant. Given that cystoscopy has a sensitivity of >98% to diagnose bladder cancer, a positive urinary cytology for malignancy is more likely to reflect a false‐positive than a missed tumour on cystoscopy 30.

Under ideal conditions, CTU has been shown to achieve a sensitivity of 95% and an NPV of 98%, suggesting that CTU can be used as a form of triage to refer patients directly for rigid cystoscopy where transurethral resection of bladder tumour can be performed, bypassing the need for flexible cystoscopy 30. While we did not find such a high sensitivity and NPV for both CTU and RBUS for the detection of cancer in our patient cohort, we found that the combination of urinary cytology with imaging resulted in an improved sensitivity for the detection of cancer, but this improvement was not sufficient to replace cystoscopy 12.

Although urinary cytology improves the detection rate of cancer when combined with imaging, this increase in diagnostic performance is at the expense of the risk of false‐positives. In the present cohort, 22 patients (4.3%) had a positive cytology result despite a normal cystoscopy and upper tract imaging. None of these patients had a subsequent diagnosis of cancer. A substantial number of patients underwent further invasive tests, such as ureteroscopy with/without ureteric urine sampling or an interval cystoscopy, while others underwent a repeat urinary cytology, which was reassuring. All of these tests were triggered by a false‐positive cytology result which led to costly, unnecessary tests, carrying additional risk and contributing to patient anxiety.

Limitations of the present study include the difference in methods used for the collection of urine and processing for cytopathological analysis. The classification of positive cytological analysis may also have differed among cytopathologists. In addition, there was no central review of cytology results; however, these results reflect the diagnostic ability of urinary cytology throughout the UK, which will inform policy‐makers. There were 47 cancers in the present series, which was low. A larger series may suggest a small benefit for urinary cytology in patients with a normal cystoscopy and imaging, as previously reported by others 13, 14. We acknowledge that urinary cytology may test positive because of a cancer anticipatory effect 31. While we do not have long‐term follow‐up data for patients where cytology was positive with a normal cystoscopy and imaging, these patients were followed up till discharged from urology care.

In conclusion, urine cytology will miss a significant number of muscle‐invasive bladder cancer and high‐risk non‐muscle‐invasive disease. Our results suggest that urinary cytology should not be routinely performed as part of haematuria investigations. The role of urinary cytology in select cases should be considered in the context of the impact of a false‐positive result leading to further potentially invasive tests conducted under general anaesthesia. Future larger, prospective, observational studies are required to validate these findings. Until urinary biomarkers with a high diagnostic accuracy have been independently validated, cystoscopy and upper tract imaging will remain the cornerstone test for patients with haematuria 32.

## Conflict of Interest

None declared.

## Appendix 1

### DETECT I collaborators

Participating centres and investigators (*principle investigators at each centre):

WS Tan, P Khetrapal, AN Sridhar, BW Lamb, F Ocampo, H McBain, JD Kelly* (UCLH), K Baillie, K Middleton, J Cresswell, D Watson* (James Cook University Hospital), H Knight, S Maher, A Rane* (East Surrey Hospital), B Pathmanathan, A Harmathova, G Hellawell* (London North West University Healthcare), S Pelluri, J Pati* (Homerton Hospital), A Cossons, C Scott, S Madaan* (Darent Valley Hospital), S Bradfield, N Wakeford, H Mostafid* (Royal Surrey County Hospital) A Dann, J Cook, M Cornwell, R Mills* (Norfolk & Norwich University Hospital) S Thomas, S Reyner, G Vallejera, P Adeniran, S Masood* (Medway Maritime Hospital), N Whotton, K Dent, S Pearson, J Hatton, M Newton, E Hheeney, K Green, S Evans, M Rogers* (Northern Lincolnshire & Goole NHS Foundation Trust), K Gupwell, S Whiteley, A Brown, J McGrath* (Royal Devon and Exeter Hospital), N Lunt, P Hill, A Sinclair* (Macclesfield Hospital), A Paredes‐Guerra, B Holbrook, E Ong* (North Devon District Hospital), H Wardle, D Wilson, A Bayles* (University Hospital of North Tees), R Fennelly, M Tribbeck, K Ames, M Davies* (Salisbury District Hospital), J A Taylor, E Edmunds, J Moore* (East Sussex Healthcare NHS Trust), S Mckinley, T Nolan, A Speed, A Tunnicliff, G Fossey, A Williams, M George, I Hutchins, R Einosas, A Richards, A Henderson* (Maidstone Hospital), B Appleby, L Kehoe, L Gladwell, S Drakeley, J A Davies, R Krishnan* (Kent & Canterbury Hospital), H Roberts, C Main, S Jain* (St James's University Hospital), J Dumville, N Wilkinson, J Taylor, F Thomas* (Doncaster Royal Infirmary), K Goulden, C Vinod, E Green* (Salford Royal Hospital), C Waymont, J Rogers, A Grant, V Carter, H Heap, C Lomas, P Cooke* (New Cross Hospital), L Scarratt, T Hodgkiss, D Johnstone, J Johnson, J Allsop, J Rothwell, K Connolly, J Cherian* (The Pennine Acute Hospitals NHS Trust), S Ridgway, M Coulding, H Savill, J Mccormick, M Clark, G Collins* (Tameside General Hospital), K Jewers, S Keith, G Bowen, J Hargreaves, K Riley, S Srirangam* (East Lancashire Hospitals NHS Trust), A Rees, S Williams, S Dukes, A Goffe* (Dorset County Hospital), L Dawson*, R Mistry, J Chadwick, S Cocks, R Hull, A Loftus (Royal Bolton Hospital), Y Baird, S Moore, S Greenslade, J Margalef, I Chadbourn, M Harris, J Hicks* (Western Sussex Hospitals NHS Foundation Trust), P Clitheroe, S Connolly, S Hodgkinson, H Haydock, A Sinclair* (Stepping Hill Hospital), E Storr, L Cogley, S Natale* (Derriford Hospital), W Lovegrove, K Slack, D Nash, K Smith* (King's Mill Hospital), J Walsh, A M Guerdette, M Hill, D Payne* (Kettering General Hospital), B Taylor, E Sinclair, M Perry, M Debbarma* (Pinderfields Hospital), D Hewitt, R Sriram* (University Hospitals Coventry), A Power, J Cannon, L Devereaux, A Thompson* (Royal Albert Edward Infirmary), K Atkinson, L Royle, J Madine, K MacLean* (Royal Cornwall Hospital).
